# Spiral Fracture in Young Infant Causing a Diagnostic Dilemma: Nutritional Rickets versus Child Abuse

**DOI:** 10.1155/2017/7213629

**Published:** 2017-09-19

**Authors:** Sonia Kaushal, Manish Raisingani, Raphael David, Bina Shah

**Affiliations:** New York University School of Medicine, New York, NY, USA

## Abstract

Fractures are uncommon in young, nonambulatory infants. The differential diagnosis includes nonaccidental injury (NAI) and metabolic bone disease, including rickets. While rickets typically present after six months of age, multiple cases have been reported in younger infants. We report a case of an 11-week-old male infant who presented with a spiral fracture of the humerus and no radiologic evidence of rickets. A detailed psychosocial assessment failed to reveal any risk factors for NAI. The patient had elevated alkaline phosphatase and PTH with low 25 hydroxyvitamin D and 1,25 dihydroxyvitamin D levels. Additionally, the mother was noncompliant with prenatal vitamins, exclusively breastfeeding without vitamin D supplementation, and had markedly low vitamin D levels 15 weeks postpartum. The biochemical data and history were consistent with rickets. Given the diagnostic dilemma, the working diagnosis was rickets and the patient was started on ergocalciferol with subsequent normalization of his laboratory values and healing of the fracture. These findings are consistent with nutritional rickets largely due to maternal-fetal hypovitaminosis D. This case highlights that in young infants rickets should be considered even in the absence of positive radiologic findings. Additionally, it illustrates the importance of maintaining adequate vitamin D supplementation during pregnancy and early infancy.

## 1. Introduction

Fractures in young infants are uncommon, particularly in nonambulatory children younger than eight months of age [1]. The differential diagnosis for fractures that do occur includes birth trauma, nonaccidental injury (NAI), and pathologic fractures. It is important to recognize that there are a number of rare, inherited, and acquired metabolic bone diseases ranging from osteogenesis imperfecta to hypophosphatasia, scurvy, and even rickets that can lead to the development of fragile bones in infancy [2]. Notably, nutritional rickets have reemerged as a public health concern despite a previously low incidence in many countries [3, 4].

Fractures are the second most common presentation of child abuse after soft tissue bruising and burns [5, 6]. NAI is responsible for most fractures in children less than two years of age with rates ranging from 31% to 60% [7, 8]. Fractures that have been identified as highly specific for abuse include fractures of the metaphysis, spinous process of vertebrae, posterior ribs, and sternum. While less specific than the fractures listed above, long bone fractures, including both spiral and transverse, are also commonly seen in child abuse.

Classically, nutritional rickets present between six and 30 months of age. Despite this, multiple cases have been reported with infants less than six months of age [9, 10]. Most of these infants presented with symptoms of hypocalcaemia, biochemical, and/or radiologic signs of rickets and a few cases that had evidence of fracture [11, 12]. The overall resurgence of nutritional rickets has been largely attributed to maternal hypovitaminosis D, an important risk factor.

Here we report an unusual case of an 11-week-old infant who presented with a spiral fracture of the humerus that caused a diagnostic dilemma of NAI versus nutritional rickets.

## 2. Case Presentation 

An 11-week-old boy was born at 36.3 weeks via spontaneous vaginal delivery with a birth weight of 3.2 kg and normal APGAR scores. He had an uncomplicated three-day NICU stay for mild respiratory support and feeding assistance and was discharged home where he continued to grow and develop appropriately. At 11 weeks of age, the parents noticed that the infant was not moving his right arm while crying. They brought the infant to the pediatrician's office early the next morning where it was suspected that he had a radial head subluxation. The pediatrician attempted to reduce it twice by twisting the baby's arm. However, he was not able to hear or feel it “pop” back into place and referred the patient to our hospital for orthopedic evaluation.

**Figure 1:**
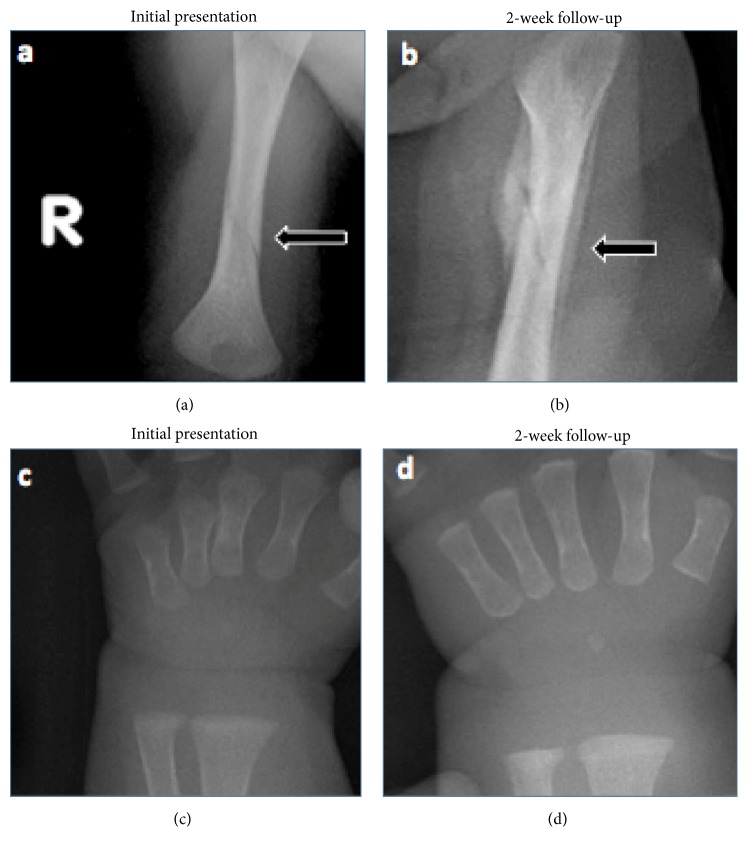
(a) Radiograph of the right upper extremity with obliquely orientated lucency through the distal humeral metadiaphysis with associated soft tissue swelling that is consistent with a nondisplaced spiral fracture. (b) Follow-up radiograph of right upper extremity with ongoing interval healing of the right spiral fracture of the middistal diaphysis of the right humerus with surrounding periosteal new bone/callus formation. (c, d) Radiographs of the right wrist at initial presentation and two-week follow-up with no signs of cupping or fraying.

Upon admission to the hospital, a physical exam revealed that the baby's right upper extremity was warm and swollen with grimace on manipulation. The remainder of the examination was unremarkable. A radiograph revealed a right distal humeral nondisplaced spiral fracture with soft tissue swelling as seen in Figure 1(a). Given the concern for NAI, a complete physical exam to rule out signs of abuse was negative for retinal hemorrhage, burns, purpura, or ecchymosis. In addition, a skeletal survey and head CT ruled out any additional fractures or signs of intracranial hemorrhage. An in-depth psychosocial assessment revealed that the infant was the third of three children living at home with his parents and two older sisters (aged 3 years and 19 months). The parents were the primary caretakers and all family members were healthy. Ultimately, it was decided that the family was reliable and the overall presentation was not consistent with child abuse.

The workup then shifted to identifying an underlying metabolic bone disease. Biochemical investigation revealed normal levels of Ca (10 mg/dL) and phosphate (5.1 mg/dL), elevated alkaline phosphatase (595 U/L) and PTH (120 pg/ml), and low levels of 25 hydroxyvitamin D (<13 ng/ml) and 1,25 dihydroxyvitamin D (13 pg/ml) as seen in Table 1. A repeat physical exam and X-rays failed to show any craniotabes, widened sutures, rachitic rosary, or enlarged wrists. Further questioning revealed that the mother had inconsistently been taking prenatal vitamins during her pregnancy, was exclusively breastfeeding without any vitamin D supplementation, and had limited her own dairy intake in an effort to make the patient less colicky. The findings at this time were most consistent with rickets.

## 3. Outcome

The patient was initially started on 2000 IU of ergocalciferol daily for two weeks followed by 800 IU per day as maintenance therapy. He responded well to therapy, with follow-up radiographs at two weeks showing interval healing of the fracture as seen in Figure 1(b). His biochemical tests were improving and normalized 15 weeks later: alkaline phosphatase (347 U/L), PTH (13.2 pg/ml), 25 hydroxyvitamin D (36.5 ng/ml), and 1,25 dihydroxyvitamin D (77 pg/ml) as seen in Table 1. Maternal biochemical investigation at 15 weeks postpartum revealed low 25 hydroxyvitamin D (8.7 ng/ml) and 1,25 dihydroxyvitamin D (22.1 pg/ml). This confirmed the suspicion that the patient had rickets that were caused by maternal-fetal-neonatal hypovitaminosis D.

## 4. Discussion

Nonaccidental injury (NAI) in our patient was ruled out by a comprehensive analysis conducted by a multidisciplinary team comprised of an attending pediatrician, the house staff, the child protection team, social worker, and ancillary staff. The psychosocial assessment in conjunction with lack of additional physical findings ruled out child abuse. The biochemical workup was consistent with rickets, a diagnosis supported by the maternal history of noncompliance with prenatal vitamins, exclusive breastfeeding without vitamin D supplementation, and confirmed maternal hypovitaminosis D. The patient's response to treatment as demonstrated by normalization of biochemical markers and healing of the fracture served as further support for this diagnosis. Despite the evidence listed above there are two factors that made this case unusual. The patient was younger than most, with the majority of patients diagnosed between the ages of six and 30 months. And lastly, our patient lacked the radiographic changes that are typically seen in rickets. Also, the initial alkaline phosphatase level was only mildly elevated. Previous studies have also shown that alkaline phosphatase may not be very high in early or subclinical rickets and may not always be correlated with the severity of rickets [13–15].

Typically, the diagnosis of rickets is confirmed with radiographic evidence which manifests as widening and irregularity of the physes with fraying and cupping of the metaphyses. These metaphyseal changes are due to impaired apoptosis of the hypertrophied chondrocyte layer and are caused by low phosphate levels in the physics [16, 17]. Additionally, secondary hyperparathyroidism causes increased bone turnover via osteoclast meditated reabsorption and this process accounts for osteopenia that is seen in the diaphysis. There have been cases of infants less than 6 months of age where patients either completely lacked the above-mentioned radiologic findings or had subtle radiologic signs suggestive of rickets [18, 19]. Although not detected by plain radiography, we postulate that our patient had a relatively higher degree of osteopenia of the diaphysis as opposed to metaphyseal changes. Thus, his bone was prone to fracture with minimal trauma, while lacking the classic metaphyseal findings.

There have also been recent studies suggesting that metaphyseal lesions which are believed to be associated with child abuse may also be associated with metabolic bone diseases [20]. Classic metaphyseal lesions are believed to consist of hypertrophic chondrocytes which results from lack of blood supply due to injury and no subsequent resorption of terminal chondrocytes [20, 21]. A similar pattern may also be seen in the active stages of rickets because of the inability of vascular invasion and subsequent resorption of terminal chondrocytes due to absence of mineralized matrix [20, 22]. Hence, metabolic bone disease should always be considered in fractures believed to be consistent with child abuse as they could have similar radiological and histological findings.

Based on our patient's overall presentation, his rickets were likely due to maternal hypovitaminosis D. Since breast milk contains insufficient quantities of vitamin D (14–25 IU/L) and infants are rarely exposed to direct sunlight, infants not supplemented with vitamin D depend largely on fetal stores to maintain adequate levels of vitamin D [23, 24]. In order to better understand how maternal levels impact infants, we conducted a literature review on the prevalence of maternal-fetal hypovitaminosis D. We defined vitamin D deficiency to be <20 ng/ml [25] and listed our results in Table 2. Through our analysis of 1020 healthy mother-infant pairs we found that plasma levels of 25 hydroxyvitamin D are closely related between mothers and their infants [26–31]. When looking specifically at infants with symptomatic vitamin D deficiency, we found that 95% of mothers also had hypovitaminosis as seen in Table 2 [9, 10, 32–37]. These results are consistent with a study by Hoogenboezem that found that total vitamin D metabolites in maternal-fetal plasma are closely related at birth and 70% of unsupplemented infants have low levels of 25 hydroxyvitamin D by eight weeks of life [38]. Together these findings suggest that vitamin D levels at birth are dependent on maternal levels and that the majority of fetal stores become depleted by eight weeks of life. This provides an explanation to the timeline of our patient's presentation at 11 weeks.

In conclusion, spiral fractures while commonly caused by NAI, can also rarely be caused by rickets. This should be considered when clinical data is suggestive of vitamin D deficiency and a history of maternal hypovitaminosis D exists. Additionally, the lasting impact of maternal vitamin D levels on young infants cannot be ignored, and improved surveillance of vitamin D levels should occur throughout pregnancy.

## Figures and Tables

**Table 1:** Infant and maternal biochemical data.

Age of infant (months)	Ca mg/dL	PO_4_ mg/dL	AlkP U/L	PTH pg/mL	25(OH)D ng/mL	1,25(OH)D pg/mL	Daily treatment with ergocalciferol (IU)
	Reference values						
	8.3–10.3	4.5–6.7	70–350	15–75	>20.0	15–75	
*Infant's biochemical data*							
Birth	7.6	7.0	—	—	—	—	—
2.5^*∗*^	10	5.1	**595**	**120**	**<13**	**13**	2,000
3	10.2	5.6	**715**	**<6.3**	**17.1**	209	800
4	10.0	—	**412**	27.9	28.4	—	800
7	10.3	6.0	**347**	13.2	36.5	77	800

*Maternal biochemical data*							
3	9.5	4.1	95	—	**8.7**	**22.1**	—

^*∗*^Initial presentation.

**Table 2:** Literature review of healthy infants and symptomatic infants.

Reference	Number of infants	Age of infant (months)	Mean/median maternal 25(OH)D (ng/ml)	Mean/median infant 25(OH)D (ng/ml)	Percent of mothers with hypovitaminosis
Studies with normal healthy infants					
Atiq et al. (1998)	62	0–11	12.82	13.86	—
Bodnar et al. (2007)	400	0	26	21.31	—
Dawodu et al. (2003)	78	1–4	8.7	4.6	—
Gür et al. (2014)	99	0	15.1	15	—
Halicioglu et al. (2012)	258	0	11.5	11.5	—
Nicolaidou et al. (2006)	123	0	16.4	20.4	—

Studies of infants/mothers with symptomatic hypovitaminosis D					
Balasubramanian et al. (2006)	13	3.8	—	3.86	100%
Daaboul et al. (1997)	5	6.8	<8.72	<6.4	100%
Dawodu et al. (2005)	38	13.5	5.32	3.2	97.4%
Elidrissy et al. (1984)	36	10.5	5.2	9.55	97.2%
Caviglia et al. (2005)	15	2	7.81	7.01	100%
Mehrotra et al. (2010)	60	3	6.54	4.92	89%
Nozza and Rodda (2001)	31	16	—	—	90.3%
Robinson et al. (2006)	63	15.1	—	—	100%

## Abbreviations

NAI: Nonaccidental injury.
